# BC-N102 suppress breast cancer tumorigenesis by interfering with cell cycle regulatory proteins and hormonal signaling, and induction of time-course arrest of cell cycle at G1/G0 phase

**DOI:** 10.7150/ijbs.62808

**Published:** 2021-07-25

**Authors:** Bashir Lawal, Yu-Cheng Kuo, Alexander T. H. Wu, Hsu-Shan Huang

**Affiliations:** 1 PhD Program for Cancer Molecular Biology and Drug Discovery, College of Medical Science and Technology, Taipei Medical University and Academia Sinica, Taipei 11031, Taiwan; 2 Graduate Institute for Cancer Biology & Drug Discovery, College of Medical Science and Technology, Taipei Medical University, Taipei 11031, Taiwan; 3 Department of Pharmacology, School of Medicine, College of Medicine, Taipei Medical University, Taipei 11031, Taiwan; 4 School of Post-baccalaureate Chinese Medicine, College of Chinese Medicine, China Medical University, Taichung 40402, Taiwan; 5 The PhD Program of Translational Medicine, College of Medical Science and Technology, Taipei Medical University, Taipei 11031, Taiwan; 6 Clinical Research Center, Taipei Medical University Hospital, Taipei Medical University, Taipei 11031, Taiwan; 7 TMU Research Center of Cancer Translational Medicine, Taipei Medical University, Taipei 11031, Taiwan; 8 Graduate Institute of Medical Sciences, National Defense Medical Center, Taipei 11490, Taiwan; 9 School of Pharmacy, National Defense Medical Center, Taipei 11490, Taiwan; 10 PhD Program in Drug Discovery and Development Industry, College of Pharmacy, Taipei Medical University, Taipei 11031, Taiwan

**Keywords:** ER+ breast cancer, G_1_/G_0_ cell cycle arrest, cell cycle proteins, chromatin immunoprecipitation, tumor progression, hormonal signaling

## Abstract

Mechanisms of breast cancer progression and invasion, often involve alteration of hormonal signaling, and upregulation and/or activation of signal transduction pathways that input to cell cycle regulation**.** Herein, we describe a rationally designed first-in-class novel small molecule inhibitor for targeting oncogenic and hormonal signaling in ER-positive breast cancer. BC-N102 treatment exhibits dose-dependent cytotoxic effects against ER+ breast cancer cell lines. BC-N102 exhibited time course- and dose-dependent cell cycle arrest via downregulation of the estrogen receptor (ER), progesterone receptor (PR), androgen receptor (AR), phosphatidylinositol 3-kinase (PI3K), phosphorylated (p)-extracellular signal-regulated kinase (ERK), p-Akt, CDK2, and CDK4 while increasing p38 mitogen-activated protein kinase (MAPK), and mineralocorticoid receptor (MR) signaling in breast cancer cell line. In addition, we found that BC-N102 suppressed breast cancer tumorigenesis in vivo and prolonged the survival of animals. Our results suggest that the proper application of BC-N102 may be a beneficial chemotherapeutic strategy for ER+ breast cancer patients.

## Introduction

Breast cancer is an invasive breast ductal carcinoma, and the most commonly diagnosed cancer (11.7% of total cases) representing 2.3 million new cases in 2020 1, 2. With an estimated 685,000 deaths, breast cancer is the fifth leading cause of cancer mortality worldwide, in both men and women 3. Among women, breast cancer respectively accounts for 25% and 16% of new cancer cases and deaths, ranking first for incidence and mortality in more than 100 countries 3. Unfortunately, reduced access to healthcare facilities, and delays in diagnosis and treatment due to the coronavirus disease 2019 (COVID-19) pandemic may lead to a short-term drop in the cancer incidence followed by an increase in advanced-stage disease and ultimately increased mortality 4, 5

Hormone receptors including the estrogen receptor (ER), progesterone receptor (PR), androgen receptor (AR), and mineralocorticoid receptor (MR) are steroid receptors with similar hormone binding, nuclear translocation, DNA binding, and transactivation domains 6. The ER is a ligand-activated nuclear receptor that mediates the transcription of estrogen-responsive genes 7, which play pivotal roles in cell growth, differentiation, proliferation, and malignant transformation 8, and thus serve as important therapeutic targets 9-12. On the basis of the ER, breast cancer is subdivided into estrogen receptor-positive (ER+) and negative (ER-) sub-types, among which ER+ accounts for ~70% of all breast cancers (expressing ER protein), while the remaining breast cancers are ER- subtypes 13, which do not express ER. PR plays an important role in normal breast development and its over-activation is detrimental for late-stage breast cancers, providing some rationale for dual targeting of ER and PR in advanced tumors 14. AR on the other hand is present in about 80 % of breast cancers making it more commonly expressed than either ER or PR, and plays an important oncogenic role in breast cancer 15. Significant progress has been made in understanding the role of ER as a transcription factor that directly regulates expressions of target genes 16 or indirectly by mediating other transcription factors on promoter targets 16, 17.

Cell cycle progression is tightly regulated by the collective activities of cyclins, CDKs, and CDK inhibitors (CDKIs). The p27, p21, and p53 are important CDKIs that negatively regulate cell cycle progression via inhibition of the cyclin/CDKs. p27 mediate G _1_ -phase arrest of the cell cycle, via cell-cell contact and growth factors dependent mechanisms, and in response to agents that inhibit cell cycle progression 18, 19. p21 binds to and inhibits the cyclin/CDK complexes, thereby preventing RB phosphorylation and thus inhibit cell proliferation 20. p53 a known tumor suppressor gene is well associated with p21 expression and exhibited dependent mechanisms of regulating tumor progression. However, it has been demonstrated that p21 expression may also be activated by p53-independent pathways 21.

However, considering the heterogeneity of breast cancer with its multiple genetic alterations and resistance to multiple treatment modalities 22, 23, targeting a single pathway by inhibiting the activity of one component would unlikely yield reasonable outcomes in the long run 24, 25. Thus, targeting multiple oncogenic signaling networks would be preferable for the discovery and development of effective anticancer drugs 26. Consequently, human epidermal growth factor receptor 2 (HER2), mammalian target of rapamycin (mTOR), cyclin-dependent kinases (CDKs), and phosphatidylinositol 3-kinase (PI3K/p110α)/protein kinase B (PKB/Akt)/extracellular signal-regulated kinase (ERK) signaling has attracted considerable attention as potential therapeutic targets for the development of new anticancer drugs to treat breast and other human malignancies 27-29. In this report, we demonstrate that BC-N102, a novel small molecule, exhibited anticancer activity against breast cancer cell lines via interfering with cell cycle regulatory proteins and hormonal receptors signaling in vitro, and exhibited potent antitumor activity at tolerated doses in an ER+ human xenograft breast cancer model. Our results provide preclinical justification to support phase I clinical trials with BC-N102 in ER+ breast cancer patients.

## Materials and Methods

### Bioinformatics analysis of breast cancer in The Cancer Genome Atlas (TCGA) pan-cancer database

We analyzed differential gene expression profiles of CDK2/CDK4 among normal breast tissues, tumor stages, metastasis, and subtypes across the TCGA pan-cancer database using the TNMplot webserver algorithm 30. Genetic and epigenetic alteration profiles of CDK2/CDK4 and survival differences between breast cancer cohorts with differential expressions or a genetically altered status of CDK2/CDK4 across the TCGA pan-cancer database were analyzed using the cBioportal 31, GSCLite 32, and MethSurve 33 webserver algorithms, while gene expression correlations were analyzed using the TIMER2.0 resource 34.

### Cell lines and culture

A normal breast cell line (MCF-10A) and a breast cancer cell line (ER+HER2-Luminal 1; MCF-7) were obtained from American Type Culture Collection (ATCC, Manassas, VA, USA), while MDB-MB-231/ATC, HS 578T, T-47D, and MDB-MB-468 were sources from the US National Cancer Institute. Cells were cultured in Dulbecco's modified Eagle medium (DMEM) containing 25 units/mL of penicillin, 25 units/mL of streptomycin, and 10% fetal bovine serum (FBS) at 37 °C in a 5% CO_2_ and 95% humidity incubator. Culture media were replaced after 72 h, and cells were subcultured to 70%~80% confluence.

### Drugs and chemicals

The clinical drug (paclitaxel) was purchased from Selleckchem (Houston, TX, USA), while BC-N102 was synthesized through established protocols in our lab 35, 36. A 10-mM stock solution of the drugs was prepared in dimethyl sulfoxide (DMSO) and kept frozen at a temperature of -20 °C. DMEM was obtained from Gibco-Invitrogen (Grand Island, NY, USA). Other reagents and chemicals used included Cremophor EL (Sigma, St. Louis, MO, USA), dimethyl acetamide (DMA) (Sigma), estradiol cyclopentyl propionate (Estol-depot injection, Astar, Taiwan), FBS (HyClone, Logan, UT, USA), Matrigel (BD Biosciences, USA), and phosphoric acid (Wako, Japan).

### *In vitro* anticancer assay

*In vitro* anticancer activities of BC-N102 were assayed using established protocols of the sulforhodamine B (SRB) reagent 37. Approximately 5000~40,000 viable cells of the NCI's 60 human tumor cell lines representing leukemia, melanoma, non-small-cell lung cancer (NSCLC), colon, central nervous system (CNS), ovarian, renal, prostate, and breast cancer cell lines were sown in wells of 96-well plates overnight (15 h). After incubation, cells were treated with a single dose (10 μM) or multiple doses (0, 0.1, 1.0, 10, and 100 μM) and incubated for 2 days. After incubation, the medium was removed, and plates were washed thrice with phosphate-buffered saline (PBS) (1%), followed by treatment with 10% trichloroacetic acid and incubation (at -4 °C) for 1 h. The plates were washed with double-distilled (dd)H_2_O, and incubated with SRB (0.4%) for 1 h. The unbound SRB dye was removed using 1% acetic acid, and the plates were air-dried. The contents of the plates were re-dissolved in a 20 mM Tris-based solution for 15 min under constant agitation. Cell viability was determined with a spectrophotometer at a wavelength of 515 nm. Growth inhibition was calculated in relation to cells without drug treatment and the time-zero control. GI_50_ is calculated from [(Ti-Tz)/(C-Tz)] × 100 = 50, while TGI is calculated from Ti = Tz, where Tz, Ti, and C represent absorbance at time 0, after treatment and for the control respectively 38, 39.

### Chromatin immunoprecipitation (ChIP) analysis

MCF-7 cells were seeded into 10-cm Petri dishes and incubated for 24 h. After 24 h of cell incubation, the medium was replaced with phenol red-free DMEM containing 5% dextran-coated charcoal (DCC)-treated FBS, and cells were incubated for another 24 h. ChIP analyses were conducted after treatment with estrogen (17β-estradiol; Sigma Aldrich, St. Louis, MO, USA), and chromatin was immunoprecipitated with 2 µg of antibodies (Santa Cruz Biotechnology, Santa Cruz, CA, USA) against ERα as previously described 13. The CDK2, CDK4, and GSTM3 promoter regions were amplified by a polymerase chain reaction (PCR). PCR products were analyzed by agarose gel electrophoresis.

### Flow cytometric analysis

A flow cytometric analysis was conducted according to the protocol described by Gao et al. 13 Briefly, MCF-7 cells were seeded in six-well plates and incubated for 24 h. Cells were harvested, washed with cold PBS, and then fixed in ice-cold (4 °C) 75% ethanol for 12 h. After fixation, cells were washed in PBS, incubated with propidium iodide (BD Bioscience, San Jose, CA, USA) and RNase for 30 min at room temperature. Cells were then analyzed using the BD FACSVerse flow cytometer (BD Biosciences).

### Western blotting

Cell lysates from BC-N102-treated MCF-7 cell lines, were subjected to protein expression analyses using sodium dodecylsulfate polyacrylamide gel electrophoresis (SDS-PAGE) with a Mini-Protean III system (Bio-Rad, Taipei, Taiwan) and transferred onto polyvinylidene difluoride membranes using the Trans-Blot Turbo Transfer System (Bio-Rad). Membranes were incubated with primary antibodies for the PI3K (CAT: #E11-1224B, 1:1000), p-ERK (CAT: #4370, 1:1000), p-AKT (CAT: #4060, 1:1000), P38 MAPK (1:1000), CDK2 (CAT: #2546, 1:1000), CDK4 (CAT: #2906, 1:1000), cyclin D1 (CAT: #2926, 1:1000), p-CDC35C (1:1000), p21 (CAT: #2947, 1:1000), p27 (CAT: #2552, 1:1000), p53 (CAT:2557 #1:1000), and GAPDH (CAT: #10494-1-AP 1:1000) for 24 h at 4 °C, followed by incubation with the respective secondary antibodies at room temperature for 1 h. Protein signals were detected and visualized with the aid of an enhanced chemiluminescence (ECL) detection kit, and images were captured using the UVP BioDoc-It system (Upland, CA, USA).

### Luciferase reporter gene assay

We used a mouse mammary tumor virus (MMTV)-Luc reporter to examine the effect of BC-N102 on PR, AR, and MR activity. This reporter contains a degenerate glucocorticoid response element that is responsive to androgens, progesterone, and glucocorticoids/aldosterone (through the AR, PR, and MR, respectively) but is not activated to any significant extent by estrogen 40. Briefly, cells were co-transfected overnight with MMTV-Luc reporter and with BC-N102, and either progesterone (P, 300 nM), dihydrotestosterone (10 nM), or mineralocorticoid (100 nM) for T = 24 h and assayed for luciferase activity as described in a previous study 41. After subtracting the blank, the luciferase values were expressed relative to negative control conditions (MMTV-Luc reporter only, no progesterone, dihydrotestosterone, or mineralocorticoid), or with the presence of the hormone.

### Maximum tolerated dose (MTD) assay

To estimate the appropriate dose level for the anticancer study, the MTD of BC-N102 was determined in BALB/c nude mice (three per group) by intraperitoneal administration of BC-N102 in a 10-day toxicity study at various concentrations; 0, 5, 10, 20, 30, and 60 mg/kg body weight (BW). The MTD is defined as the highest concentration that causes no more than a 10% BW decrement compared to an appropriate control group and produces no mortality or any external signs of toxicity that would be predicted to shorten the natural lifespan of the animal 42, 43. All animals were examined daily for signs of toxicity. At the end of 9 days, all animals were sacrificed, and their oral cavity, liver, colon, small intestine, kidneys, and stomach were examined for any abnormalities under a dissection microscope.

### *In vivo* anticancer assay in a human breast tumor xenograft model

Female nude (nu/nu) mice aged 6~7 weeks obtained from BioLasco Taiwan (Taipei City, Taiwan; under license from Charles River Laboratories) were used. Four animals were housed in an individually ventilated cage (IVC, 26.7 x 20.7 x 14.0 cm) with controlled temperature (20~24 °C), humidity (30%~70%) and a 12-h light/dark cycle. Free access to a standard lab diet (Oriental Yeast, Tokyo, Japan) and autoclaved tap water was provided. All aspects of this work including housing, experimentation, and animal disposal were performed in general accordance with the *Guide for the Care and Use of Laboratory Animals: Eighth Edition* (National Academies Press, Washington, D.C., 2011) in an AAALAC-accredited laboratory animal facility. In addition, the animal care and use protocol was reviewed and approved by the IACUC at Eurofins Panlabs Taiwan (New Taipei City, Taiwan). Viable human breast tumor cells (MCF-7, ATCC HTB-22) at 1.5 x 10^7^ cells in a 0.2-mL mixture (Matrigel: medium = 1:1) were subcutaneously implanted into the dorsal back region of the animals, and estradiol cyclopentyl propionate at 100 μg/mouse was subcutaneously injected twice a week as a supplement during the study period 44. When the tumor had reached ≥ 5 mm in diameter (denoted as day 1), the animals were randomly divided into three groups (of six animals each). The test substance, BC-N102 (dissolved in 30% DMA/20% Cremophor EL/10 mM H_3_PO_4_) at 1 mg/kg BW was administered once daily by an intraperitoneal injection for 45 days. The reference substance, paclitaxel, at 10 mg/kg BW, was intravenously injected once weekly. The tumor size, BW, and overt signs of toxicity were observed and recorded every 4 days for 45 days. The tumor volume (mm^3^) was estimated according to the formula: length x (width)^2^ x 0.5. Tumor growth inhibition (T/C) was calculated by the following formula: T/C = (Tn - T1/Cn - C1) x 100%, where C1 (Cn) is the tumor weight measured on day 1 (day n) in the control group, and T1 (Tn) is the tumor weight measured on day 1 (day n) in the treated group.

### Molecular docking of BC-N102 with CDK2 and CDK4

The Avogadro molecular builder and visualization tool vers. 1.XX (http://avogadro.cc/) was used to obtain the mol2 file of the three-dimensional (3D) structure of BC-N102, while the SDF file of palbociclib (CID: 5330286) was obtained from the PubChem database. The PDB files (3D structures) of the receptors, CDK2 (PDB; 4EK3) and CDK4 D3 (PDB; 3G33), were retrieved from the Protein Data Bank. SDF files were converted to PDB files using the PyMOL Molecular Graphics System, vers. 1.2r3pre (Schrödinger, https://pymol.org/edu/?q=educational/). All PDB files were converted into PDBQT files using AutoDock VINA (vers. 0.8) 45. Pre-docking preparation of the ligands and receptors was conducted following the protocol described in previous studies 39. Docking was conducted using AutoDock VINA software as described previously 46. Docking results were visualized using the PyMOL software and Discovery studio visualizer (vers. 19.1.0.18287, BIOVIA, San Diego, CA, USA) 47.

### Statistical analysis

All experiments were conducted with at least three replicates (*n*≥3). GraphPad Prism software (San Diego, CA, USA) was used for data analysis and visualization. Experimental data are presented as the mean ± standard deviation (SD) and were calculated using Student's *t*-test. Purity-adjusted Pearson's correlations were used to analyze gene expression correlations in breast cancer patients. All survival analyses are presented using Kaplan-Meier plots. A *p* value of <0.05 indicated a significant difference. Statistical significance was denoted as * *p*<0.05, ** *p*<0.01, and *** *p*<0.001.

## Results

### CDK2 and CDK4 are clinical biomarkers of breast cancer (BRCA) staging and therapeutic responses

**Figure 1:**
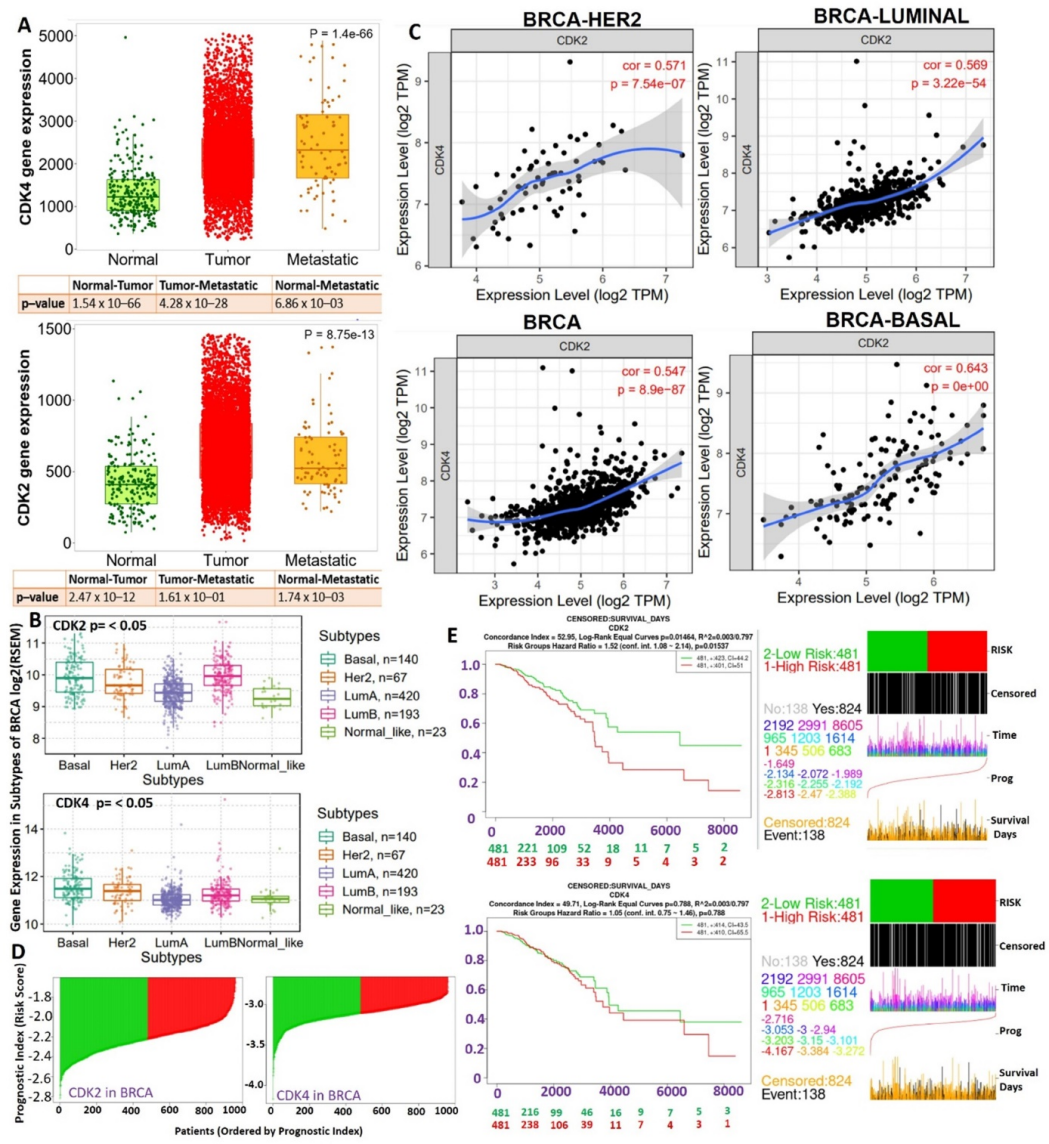
Boxplot of differential expression profiles of cyclin-dependent kinase 2 (CDK2) and CDK4 between (A) breast cancer (BRCA) tumors, normal and metastatic tissues, (B) and among breast cancer subtypes. (C) Correlation plot between CDK2 and CDK4 expressions in BRCA, basal, Her2, and luminal subtypes. (D and E) Risk scores and Kaplan-Meir plot of the differential survival analysis between breast cancer cohorts with high and cohorts with low expression profiles of CDK2 and CDK4.

To evaluate the clinical relevance of CDK2 and CDK4 in breast cancer, we analyzed CDK2- and CDK4-related clinical information of breast cancer patients from TCGA pan-cancer database. Our differential expression analysis of CDK2 and CDK4 expression profiles revealed that both CDK2 and CDK4 are overexpressed in breast cancer tumors compared to adjacent normal tissues (Figure 1A). In addition, expression profiles of CDK2 and CDK4 were associated with tumor metastasis (Figure 1A) and breast cancer subtypes, with higher expression levels in basal and Her2 subtypes than the other subtypes (Figure 1B). The expression correlation analysis revealed strong expression correlations (*r*=0.54~064, all *p*<0.001) of CDK2 and CDK4 expressions in BRCA, basal, Her2, and luminal subtypes (Figure 1C). As expected, the higher expression profiles of CDK2 and CDK4 were associated with high risk scores and shorter survival periods of the breast cancer cohorts (Figure 1D).

**Figure 2:**
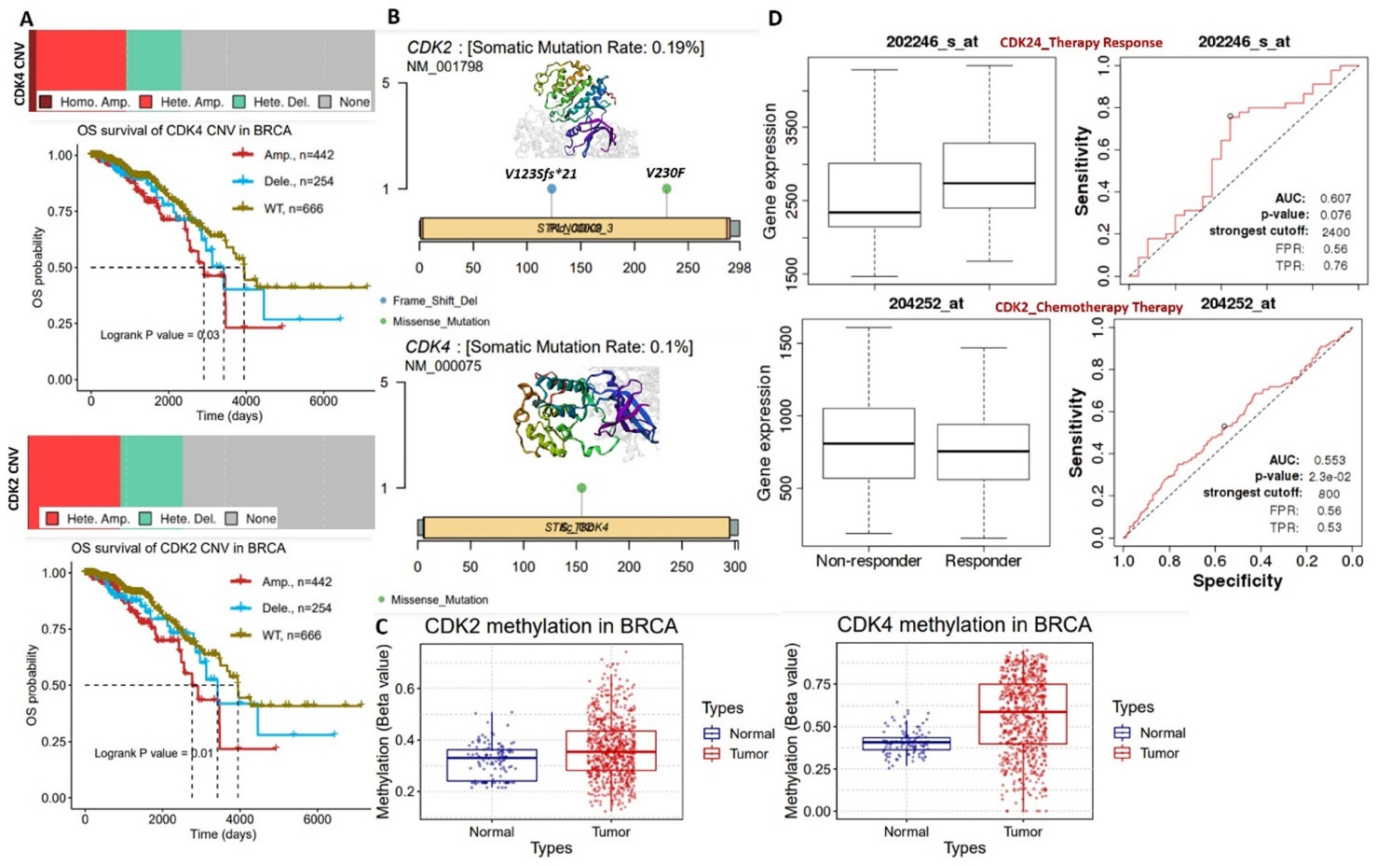
(A) Copy number variation landscape and Kaplan-Meir plot of breast cancer cohort with copy number variation (CNV) of cyclin-dependent kinase 2 (CDK2) and CDK4. (B) Somatic mutation plot of CDK2 and CDK4 in breast cancer. (C) Boxplot of differential methylation statuses of CDK2 and CDK4 between breast cancer tumors and adjacent normal tissues. (D) Gene expression and therapy response curve, showing the association of CDK2 and CDK4 expressions with non-responsiveness of breast cancer patients to therapy.

Our analysis of genetic and epigenetic alterations revealed that gene amplifications and heterozygous deletions were the most frequent copy number variations of CDK2 and CDK4 in breast cancer patients and were significantly (*p*<0.05) associated with poorer survival of breast cancer cohorts compared to cohorts with wild-type CDK2 and CDK4 (Figure 2A). In addition, CDK2 and CDK4 are hypomethylated but are less frequently mutated: CDK2 (2 cases 0.19%) and CDK2 (1 case 0.10%) in breast cancer (Figure 2B, C). Interestingly, we found that higher CDK2 and CDK4 expressions were associated with non-responsiveness of breast cancer patients to therapy (Figure 2D). Altogether, our bioinformatics analysis of clinical information from breast cancer patients strongly suggested that CDK2 and CDK4 are biomarkers of tumor progression, metastasis, and prognostic and therapy responses in breast cancer.

### BC-N102 demonstrated dose-dependent cytotoxic effects against breast cancer cell lines but no significant activities against a normal breast cell line

**Figure 3:**
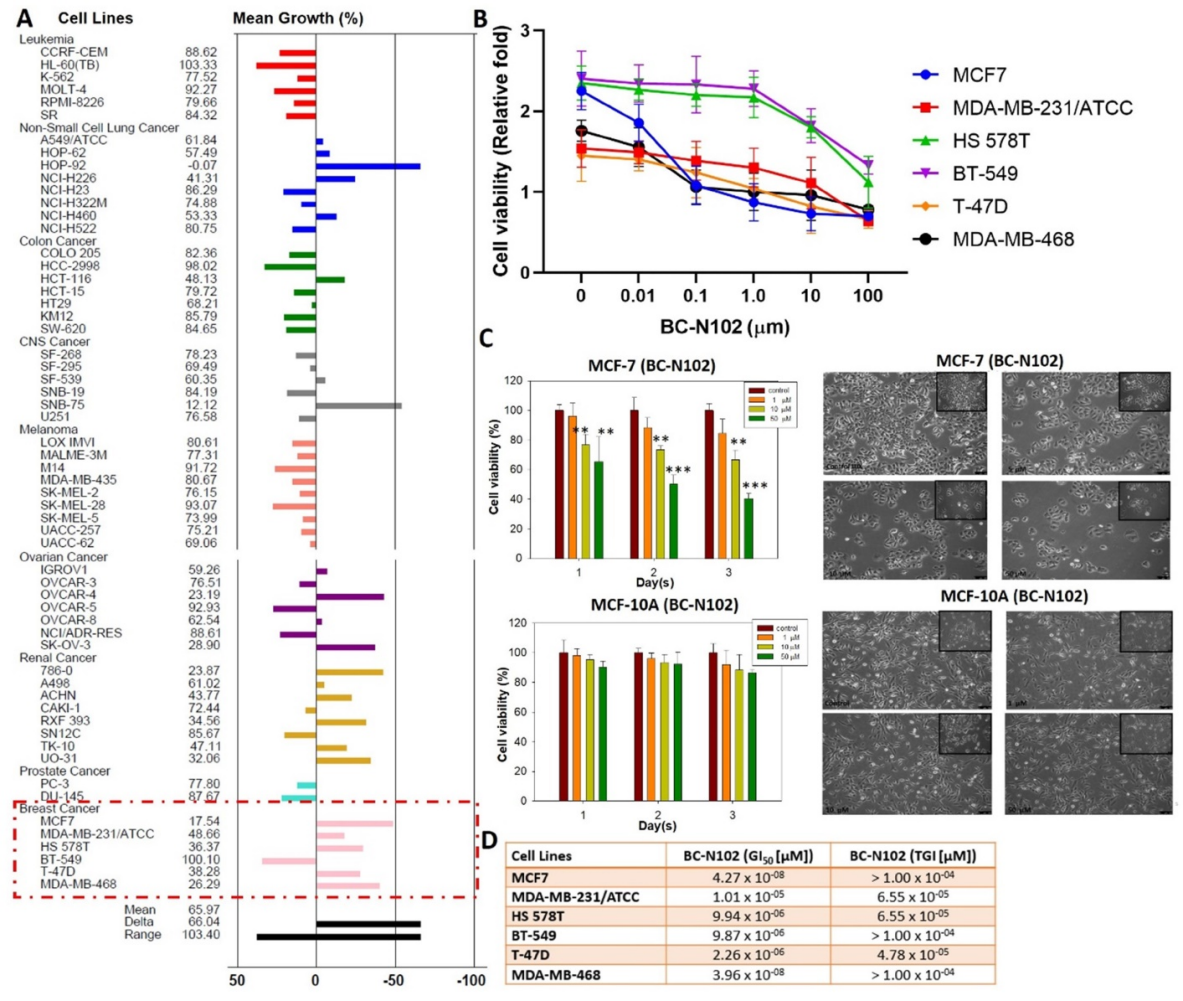
*In vitro* anticancer activities of BC-N102. Inhibitory activities of BC-N102 against panels of 60 human cancer cell lines. Each cell line was treated with a single dose of 10 μM of BC-N102. The zero point on the X-axis denotes the mean percentage of cell growth. The percentage growth of each cell line relative to the mean is represented by horizontal bars (Y axis). The right side bars indicate greater sensitivity, and left side bars indicate lower sensitivity. (B and C) Dose-response curve of BC-N102 against breast cancer cell lines and a normal breast cell line. (D) The 50% growth inhibition (GI50) and total growth inhibition (TGI) concentrations of BC-N102 against the breast cancer cell lines.

We evaluated the anticancer activities of BC-N102 against the NCI60 cell line panel of breast, prostate, renal, ovarian, colon, melanoma, CNS, leukemia, and NSCL cancers. Interestingly, we found that BC-N102 at 10 μM exhibited antiproliferative effects against all 60 cell lines (Figure 3A). In addition to the antiproliferative effects, BC-N102 demonstrated cytotoxic effects against NSCLC cell lines (A549/ATCC, HOP-62, HOP-92, NCI-H226, and NCI-H460), renal cancer cell lines (786-0, A498, ACHN, RXF393, TK-10, and UO-31), and breast cancer cell lines (MCF7, MDB-MB-231/ATC, HS 578T, T-47D, and MDB-MB-468). Furthermore, we found that BC-N102 exhibited dose-dependent cytotoxic effects against breast cancer cell lines (Figure 3B, C) with GI_50_ values of 1.01 x 10^-05^~4.27 x 10^-08^ µM and TGI values of 4.78 x 10^-05^~1.00 x 10^-05^ (Figure 3D) but demonstrated no significant antiproliferative or cytotoxic activities against a normal breast cancer cell line (MCF-10A, Figure 3C). Taken together, these findings indicated that BC-N102 has cytotoxic effects against breast cancer cell lines but no significant activities against a normal breast cell line, and could serve as a novel candidate for treating breast cancer.

### BC-N102 induced time-course arrest of the cell cycle at GO/G1 phase via interfering with the signaling of cell cycle regulatory proteins in breast cancer cell line

**Figure 4:**
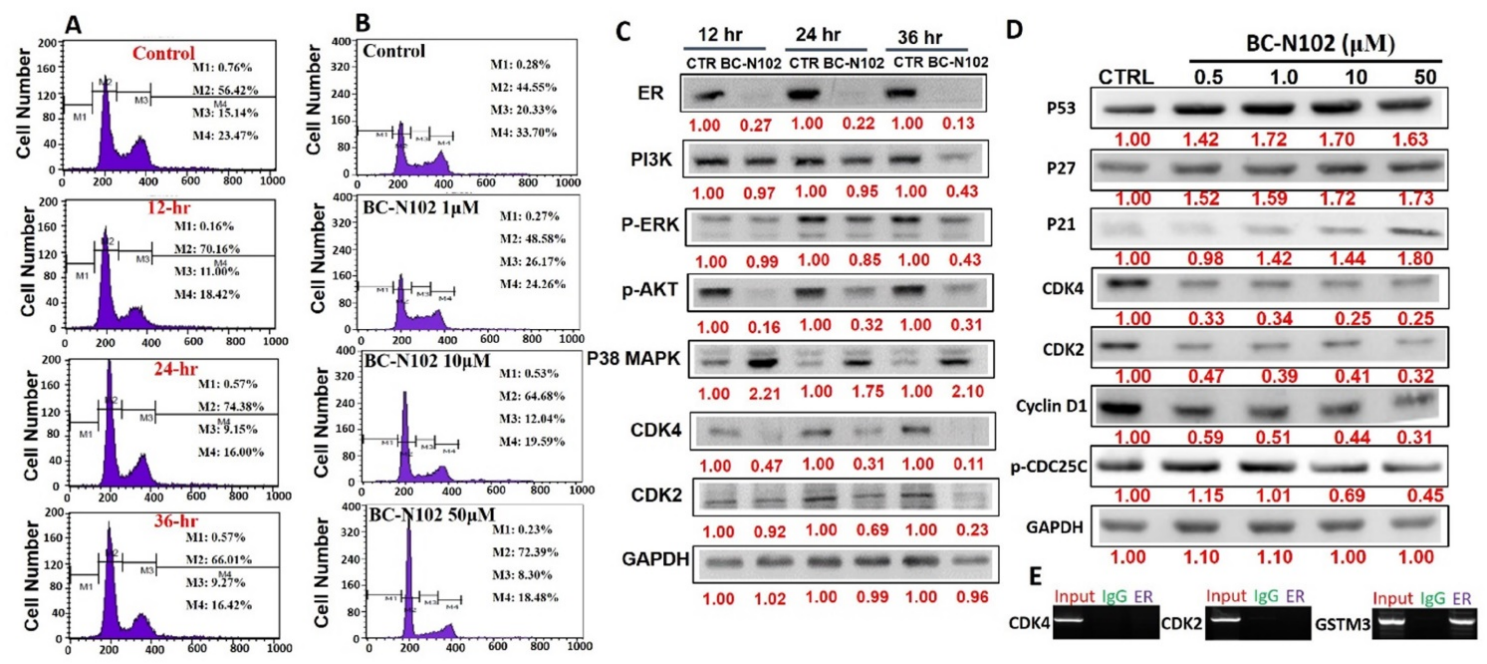
BC-N102 exhibited time course- and dose-dependent cell cycle arrest of the MCF-7 cell line***.***Flow cytometry showing the (A) time-dependent and (B) concentration-dependent effects of BC-N102 against different stages of MCF-7 cell cycle progression. (C) Western blot showing the time-dependent effect of BC-N102 treatment on the expression levels of the estrogen receptor (ER), phosphatidylinositol 3-linase (PI3K), phosphorylated (p)-extracellular signal-regulated kinase (ERK), p-Akt, p38 mitogen-activated protein kinase (MAPK), cyclin-dependent kinase 2 (CDK2), and CDK4 expressions in the MCF-7 cell line. (D). western blot showing the dose dependent effect of BC-N102 treatment on the expression levels of CDK2, CDK4, cyclin D1, phosphorylated CDC35C, p21, p27, and p53 in MCF-7 cell line. Numbers in red indicate relative expression ratios from the band quantification. (E) Chromatin Immunoprecipitation (ChIP) assay of the binding capacity of the ER for CDK2 and CDK4. The ChIP assay showed that the ER did not interact with the CDK2 or CDK4 promoter regions.

**Figure 5:**
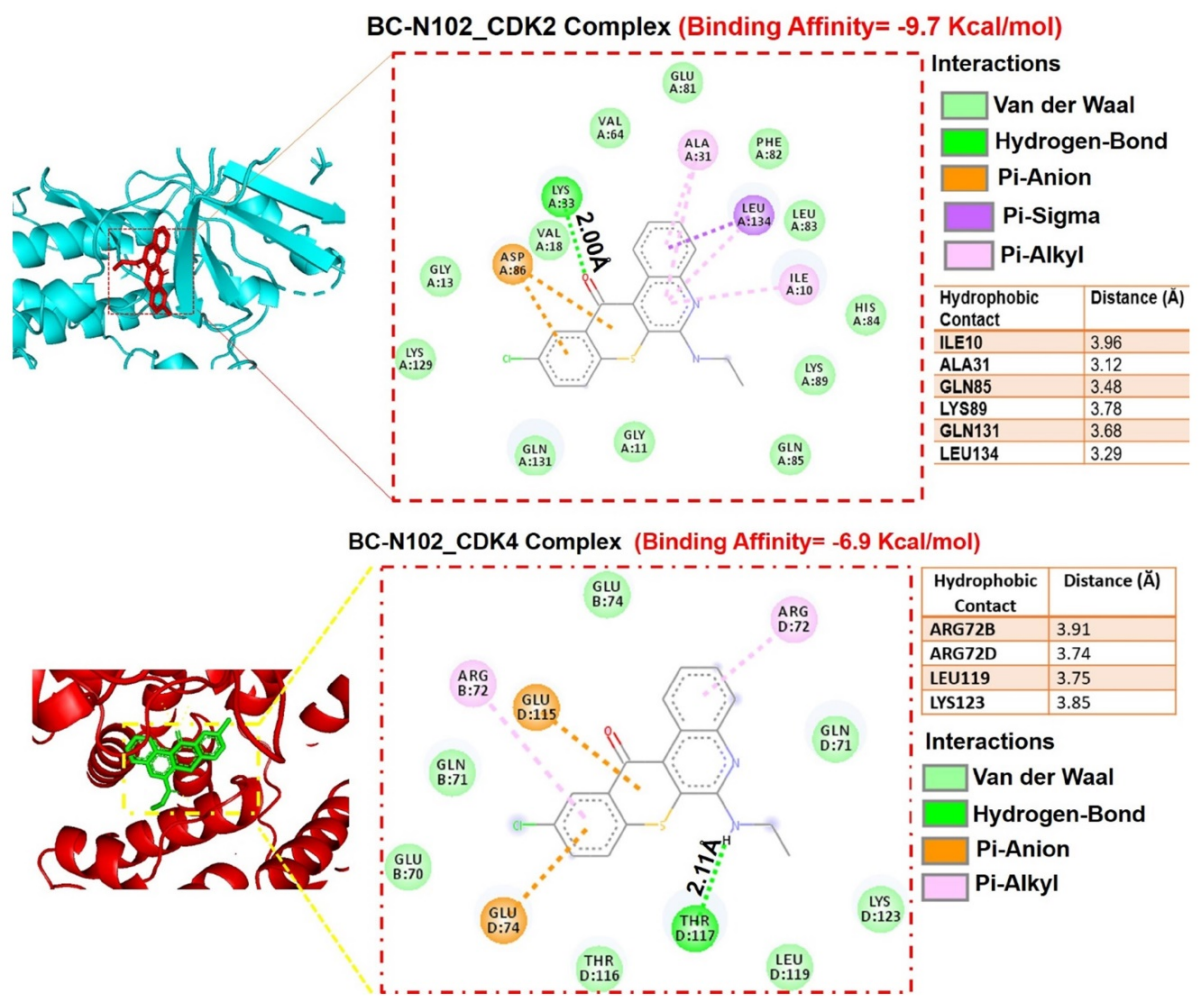
Molecular docking profiles of BC-N102 with cyclin-dependent kinase 2 (CDK2)/CDK4. (A) Two dimensional (2D) representations of ligand-receptor complexes, showing the interacting amino acid residues and types of interactions occurring between the ligand (BC-N102) and receptors (CDK2 and CDK4). The accompanying table shows the hydrophobic contacts between the ligand and receptors.

We used flow cytometry to analyze the effects of BC-N102 on different stages of MCF-7 cell cycle progression. Interestingly we found that treatment of MCF-7 cells with BC-N102 led to time-dependent arrest of the cell cycle at the G_0_/G_1_ phase with peak activities after 24 h of treatment (Figure 4A). In addition, BC-N102 treatment also led to dose-dependent arrest of the MCF-7 cell cycle at the G_0_/G_1_ phase. Cell arrest at this phase increased to 0%, 48.55%, 64.68%, and 72.39% with 0, 1, 10, and 50 μM of BC-N102, respectively (Figure 4B). The roles of the ER and cell cycle-related genes in cell cycle regulation were reported in several studies. Therefore, we wondered whether effects of BC-N102 on cell cycle progression and cell proliferation were linked to downregulation of these genes. We performed a Western blot analysis and found that treatment of MCF-7 cells with BC-N102 led to a time course-dependent downregulation of the ER, PI3K, p-ERK, p-Akt, p38 MAPK, CDK2, and CDK4 while increasing p38 MAPK expression in the MCF-7 cell line (Figure 4C). In addition, BC-N102 exhibited dose-dependent downregulation of CDK2, CDK4, cyclin D1, and phosphorylated CDC35C while increasing the expression levels of cell cycle regulatory proteins (p21, p27, and p53, Figure 4D). It is already known that the ER is a transcription factor that binds to promoters of estrogen-responsive target genes 48, 49. To investigate whether the downregulation of CDK2 and CDK4 is a result of the effect of BC-N102, we evaluated whether the ER directly regulates these cell cycle-related genes by binding to their promoter regions. Our ChIP assay showed that the ER did not interact with the CDK2 or CDK4 promoter region, suggesting that BC-N102 downregulated the expression of CDK2 and CDK4 independent of ER positivity in breast cancer cell lines (Figure 4E). Moreover, our molecular docking study revealed robust interactions of BC-N102 with CDK2 and CDK4 binding cavities with binding affinities (ΔG) of -9.7 and -6.9 kcal/mol respectively. The BC-N102 bound with CDK2 and CDK4 by several hydrogen bonding, pi-interactions, hydrophobic contact, and several van der wall forces (Figure 5).

### BC-N102 interfered with ligand-mediated and ligand-independent activation of hormonal receptors signaling in breast cancer

**Figure 6:**
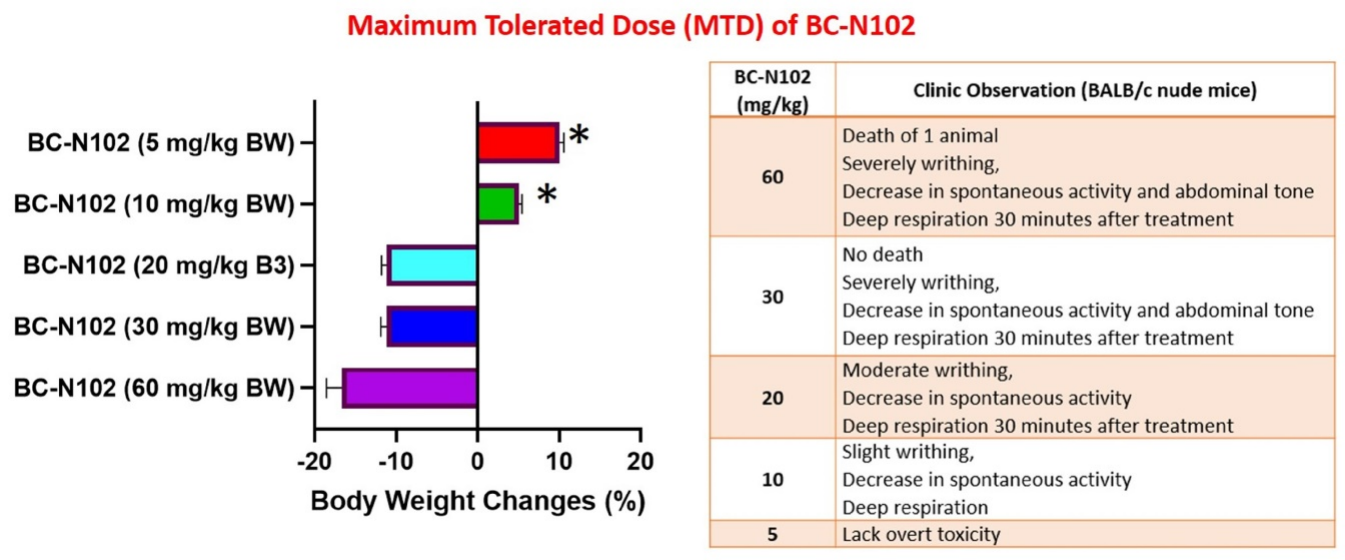
Clinical observations and body weight changes of mice treated with various concentrations of BC-N102 in the maximum tolerated dose (MTD) study.

We investigated the dose dependence effects of BC-N102 on PR, AR, and MR signaling pathways using the luciferase reporter gene assay in T47D cells. These studies revealed that BC-N102 produced a dose-dependent inhibition of PR activity in the absence of progesterone. In addition, BC-N102 produced dose-dependent inhibition of the progesterone-stimulated PR activity in a dose-dependent manner (Figure 6A). Similarly, BC-N102 caused dose-dependent inhibition of AR activity in the presence or absence of DHT (Figure 6B). The maximum inhibitory activities of BC-N102 on AR and PR occurred at a concentration of 20 μM. Our results also demonstrated that in the absence of aldosterone, BC-N102 mediated increase inhibition of MR activity with increasing concentrations from 1.25, 2.5, and 7.5 μM, however, with further increase in BC-N102 concentrations, MR activity became activated. Similarly, in the presence of aldosterone, BC-N102 results in a dose-dependent induction of aldosterone-mediated MR activity in a dose-dependent manner (Figure 6C). We evaluated differential expression profiles of PR, AR, and MR genes between tumor samples from TCGA breast cancer patients and adjacent normal tissue and found that PR and AR were over-expressed while MR gene is under-expressed in breast cancer patients compared to normal tissue (Figure 6D). Altogether, our results indicate that BC-N102 exhibited its therapeutic effect via suppression of PR and AR signaling while mediating the activation of MR signaling irrespective of the presence or absence of the receptor's ligand in the breast cancer cell line.

### *In vivo* maximum tolerated dose (MTD) study

**Figure 7:**
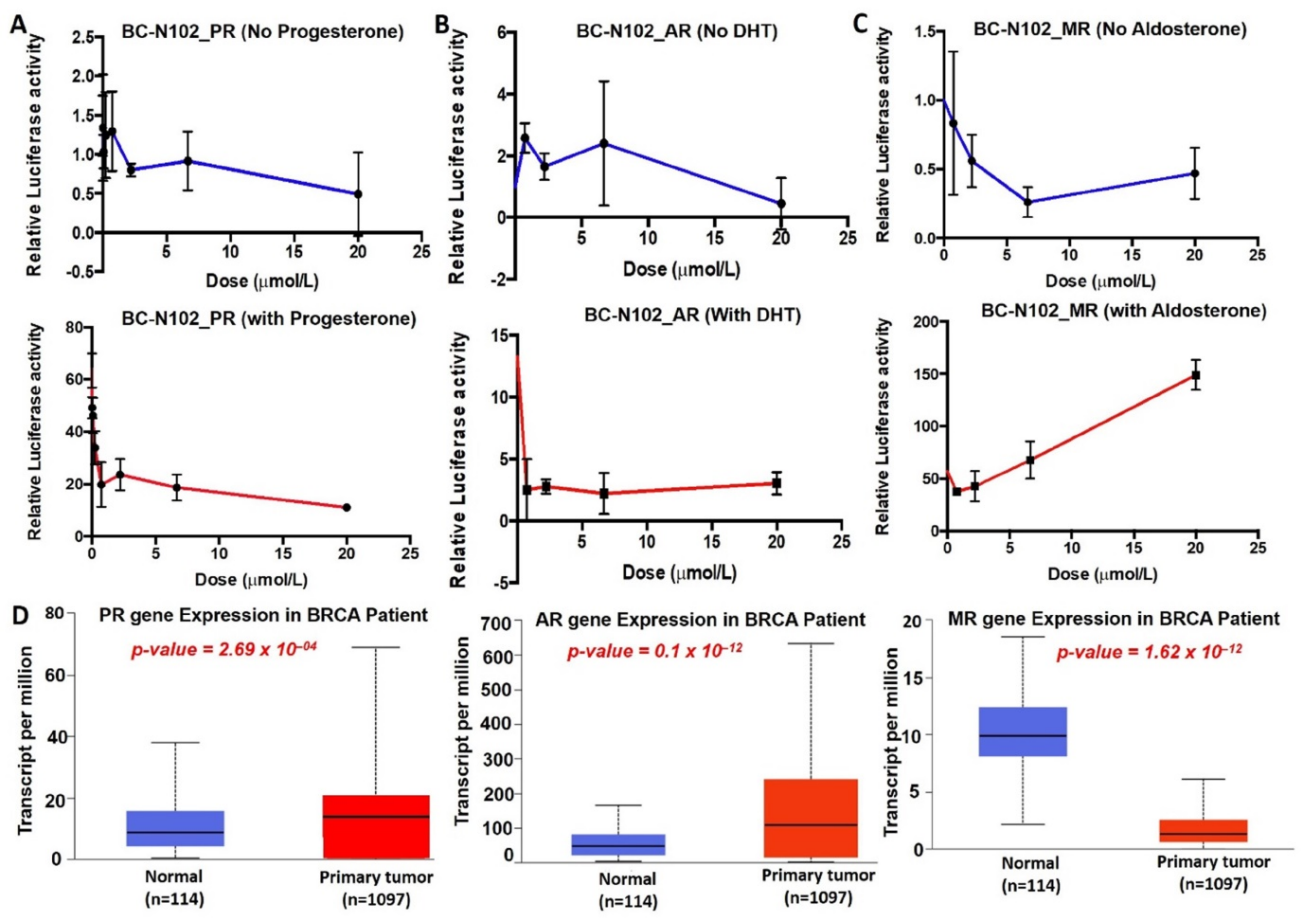
** BC-N102 interfered with the ligand-mediated and ligand-independent activation of hormonal receptors signaling in breast cancer.** Dose dependence plot of the effects of BC-N102 on (A) progesterone receptor (PR), (B) androgen receptor (AR), and (C) mineralocorticoid receptor (MR) signaling pathways in the absence (upper panel) and presence of progesterone, dihydrotestosterone, and aldosterone respectively (lower panel) respectively using the luciferase reporter gene assay in T47D cells. (D) Differential expression profile of PR, AR, and MR genes between tumor samples from TCGA breast cancer patients and adjacent normal tissue.

We examined the MTD of BC-N1025, after a single i.v. injection into BALB/c nude mice. The MTD was estimated based on the threshold at which all animals survived with no more than 10% BW loss. We found that all mice treated with 5, 10, and 20, mg/kg BW tolerated the drug. Mice treated with 5 mg/kg BW of BC-N102 were completely devoid of toxicity and showed an increased percentage of BW gain after 2 weeks. During the observation period, none of the mice exhibited weight loss of >10%, and no deterioration in health was observed in mice treated with BC-N102 at up to 20 mg/kg BW. However, decreases in spontaneous activity, writhing, and deep respiration of the animals were observed at 30 min after administration. In contrast, high loss of BW (>10%) in mice treated with 30 and 60 mg/kg BW and death of one animal treated with 60 mg/kg BW were observed (Figure 7).

### BC-N102 suppressed breast cancer tumorigenesis and prolonged survival of animals

**Figure 8:**
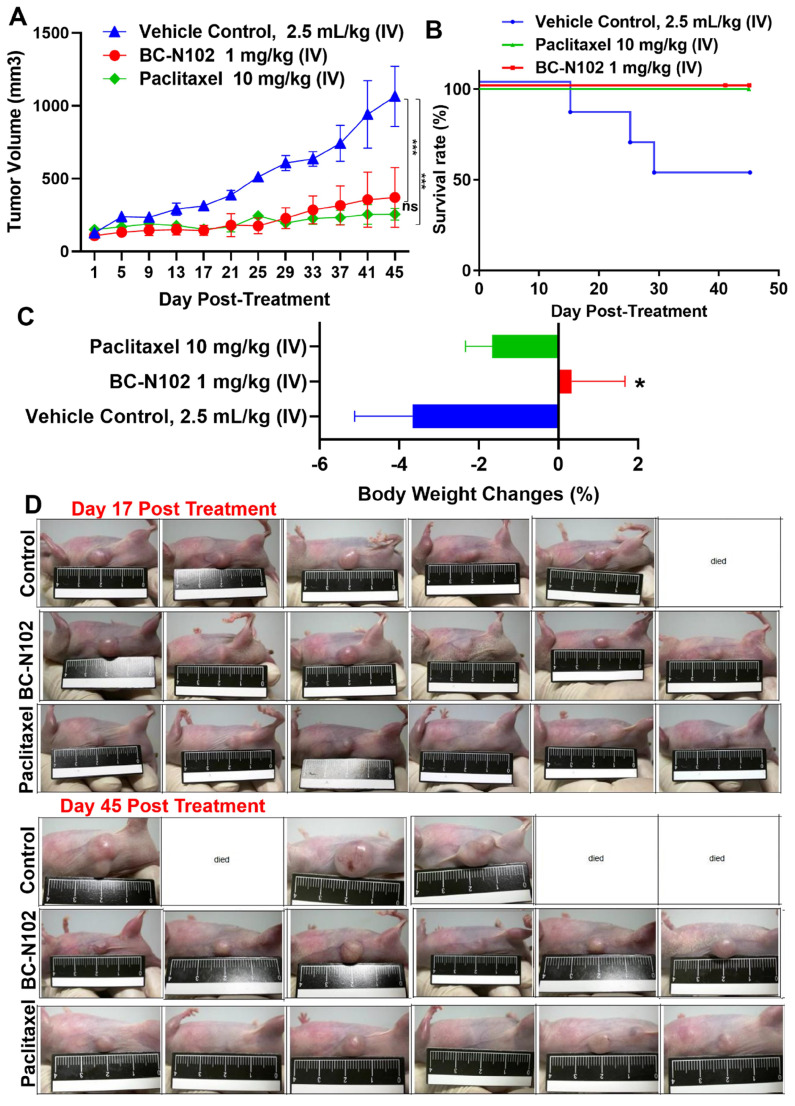
BC-N102 suppressed MCF-7 tumorigenesis *in vivo* and prolonged the survival of animals. (A) An average tumor volume versus time curve shows that treatments with BC-N102 significantly delayed MCF-7 tumor growth and the tumor burden. (B) The Kaplan-Meier survival curve shows a higher overall survival ratio of animals treated with BC-N102 compared to control counterparts. (C) Graph of percentage body weight changes of mice; animals treated with BC-N102 showed a significant increase in the percentage body weight gain compared to the control counterparts. * *p*<0.05, *** *p*<0.001.

Following our *in vitro* studies, we evaluated the *in vivo* antitumor activities of BC-N102 against the MCF-7 cell line. Treatments with BC-N102 significantly (*p*<0.001) reduced MCF-7 tumor growth (Figure 8A) and prolonged the survival rate of animals (Figure 8B) compared to the control counterparts. In addition, BC-N102 enhanced the percentage BW gain of animals (Figure 8C). The *in vivo* therapeutic efficacy of BC-N102 was comparable to the observed therapeutic effect of paclitaxel (Figure 8A, D). However, treatment with paclitaxel could not prevent weight loss in the animals.

## Discussion

The development of targeted therapies for breast cancer is impeded by the heterogeneity of cancer cells which enhances their proliferation and invasive phenotypes and regulates their responsiveness to therapies 50, 51. In addition, the molecular targets of current therapies, such as ER/PI3K/Akt, human epidermal growth factor receptor 2 (HER2), mammalian target of rapamycin (mTOR), and CDK4/CDK6, exhibit considerable intra- and inter-patient variations in their expression levels 28, 29. In line with previous studies, our analysis of clinical data revealed that CDK2/CDK4 are overexpressed in breast cancer patients and contribute to tumor progression, metastasis, invasive phenotypes and non-responsiveness to chemotherapy. Moreover, we found that our novel tetracyclic derivative, BC-N102 suppressed tumorigenesis via inhibition of ER/Akt/PI3K/ERK/CDK2/4, and induced time-course G_0_/G_1_ cell cycle arrest of human breast cancer cells.

Our results demonstrated that BC-N102 exhibited dose-dependent suppression of breast cancer cell lines in vivo and in vitro. Our mechanistic study further revealed that BC-N102 treatment led to time-course and dose-dependent arrest of the cell cycle at G0/G1 phase with concomitant downregulation of ER, PI3K, p-ERK, p-AKT, cyclin D1, CDK2, and CDK4. Cyclin-dependent kinases play an important role in cell cycle progression and tumorigenesis in various cancers 28. In line with our observation, a number of studies have associated the inhibition of CDKs to cell cycle arrest 52, 53. Zhou et al. reported that treatment of human breast cancer cells with staurosporine causes a reduction in the expression level of CDK that accompanies a G1 cell cycle arrest 54, while Ishida et al. reported that treatment of breast cancer cell lines with herbimycin A caused cell cycle arrest-related reduction of the CDK4 and CDK6 expression levels 55. In another study, it was reported that treatment of MCF-7 breast cancer cell line with 1,25(OH)2D3 induces cell cycle arrest at G0/G1phase via inhibition of CDK2 and CDK4 56-60.

The cell cycle regulatory proteins; P53, P21, P27, respond to intrinsic or extracellular anti-mitogenic signals by binding to and inhibiting cyclin/Cdks and thereby blocking cell cycle progression 61, 62. Interestingly, our results demonstrated that treatment of MCF-7 cells with BC-N102 significantly increases the activities of these cell cycle regulatory proteins and P38 MAPK. Altogether, these findings strongly suggest that BC-N102 suppresses breast cancer proliferation via cell cycle arrest by enhancing the activities of cell cycle regulatory protein which in turn inhibit the activities of cell cycle proteins including the CDK2, CDK4 and cyclin D1, and consequently led to cell cycle arrest.

**Figure 9:**
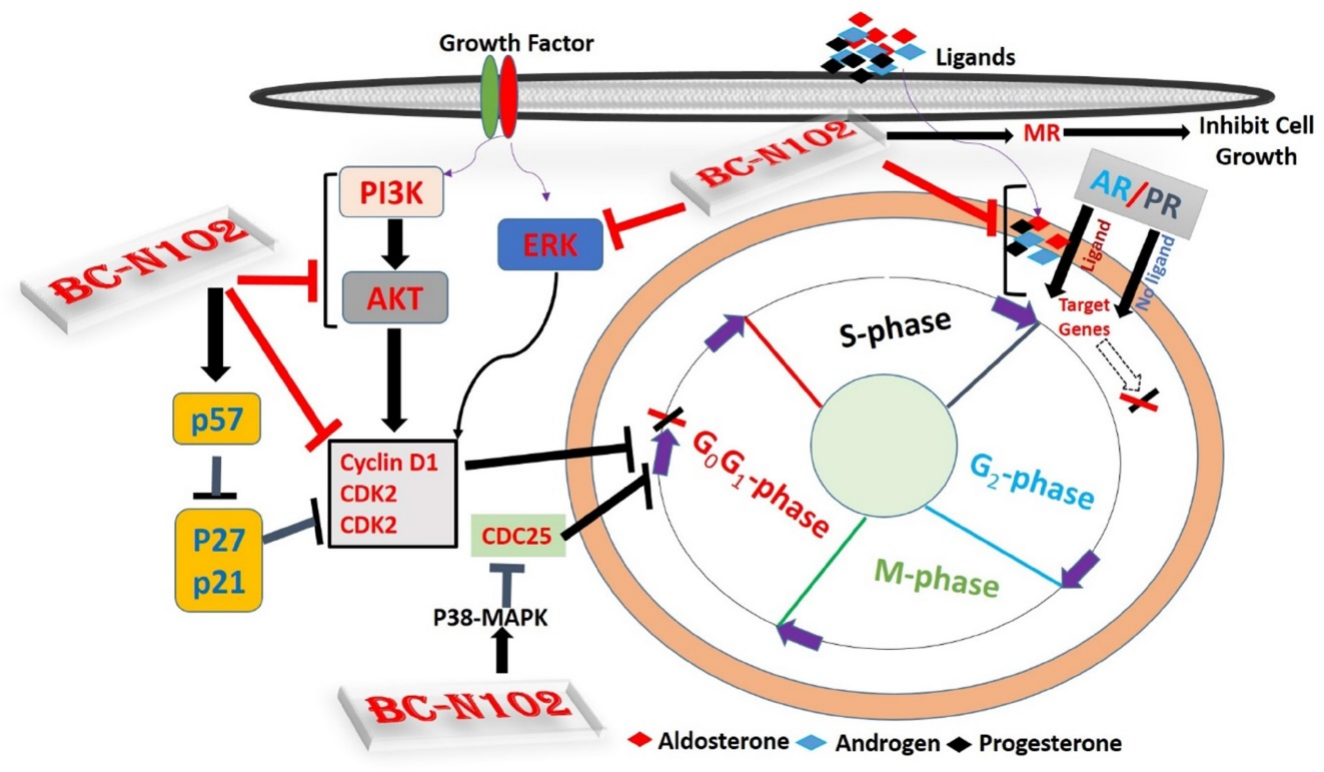
** Schematic representation of the therapeutic effect of BC-N102 in breast cancer.** BC-N102 attenuated breast cancer growth via downregulation of the estrogen receptor (ER), progesterone receptor (PR), androgen receptor (AR), phosphatidylinositol 3-kinase (PI3K), phosphorylated (p)-extracellular signal-regulated kinase (ERK), p-Akt, CDK2, and CDK4 while increasing p38 mitogen-activated protein kinase (MAPK), and mineralocorticoid receptor (MR) signaling in breast cancer cell line.

ER, AR, PR, and MR are oncogenic steroid hormones that are activated by hormone binding 63. Once activated they act as transcription factors by binding to the promoter's region of the target genes 6. However, in the absence of the ligands (hormones), these hormone receptors are activated through phosphorylation by kinases such as ERK, MAPK, PI3K, and AKT in human malignancies 64. In addition, activated receptors could also act as coactivators that mediate the activities of other transcription factors 65. Therefore, inhibition of these steroid hormone signaling would be an effective strategy of cancer therapy. Interestingly, our results revealed that BC-N102 treatment-induced dose-dependent inhibition of AR and PR in the present as well as in the absence of hormone ligands. in addition, we found that the activities of AKT, PI3K, and ERK which are known alternative activator of these hormone receptors 64, 66 were significantly downregulated by TCN-02 treatment, suggesting inhibition of both canonical (classical; ligand-mediated) and non-canonical (alternative; ligand-independent) pathways of ER, AR, and PR activation (Figure 9).

However, our observed effect of BC-N102 on mineralocorticoid receptor suggest a distinct role of mineralocorticoid in cancer development and response to therapeutic intervention. In order to understand the translational relevance of MR inhibition by BC-N102, we evaluated the differential expression profile of PR, AR, and MR genes and found that PR and AR were over-expressed while MR gene is under-expressed in breast cancer patients compared to normal tissue. Our findings are supported by a clinical study that reported that MR expression is lower in breast cancer compared to normal breast tissues and that MR is an independent predictor of metastasis-free survival in tamoxifen-treated breast cancer patients 67. In addition, studies have reported that activation of mineralocorticoid receptor induces cell adhesion and growth inhibition in breast cancer 68, 69 and another cancer type 70. This mineralocorticoid receptor-mediated tumor inhibition has been linked to inhibition of the Warburg effect 69, 70.

Previous studies revealed that ER downregulation attenuated the mitogenic effects of estrogen and induction of target genes in the MCF-7 cell line 71, 72. To investigate whether the downregulation of CDK2 and CDK4 is a result of the effects of BC-N102, we evaluated whether the ER directly regulates these cell cycle-related genes by binding to their promoter regions. Disappointedly, our ChIP assay showed that the ER did not interact with the CDK2 or CDK4 promoter regions, suggesting that BC-N102 downregulates the expression of CDK2 and CDK4 independent of the ER in breast cancer cell lines.

Patient-derived xenograft (PDX) models have translational relevance in preclinical studies of anticancer drug discovery, due to their high stability and similarity to human tumors 73. To evaluate the inhibitory effects of BC-N102 against breast cancer, a PDX model of MCF-7 was established for our *in vivo* study. Results showed that BC-N102 significantly suppressed tumor cell proliferation and prolonged the survival of animals in the PDX mice model. In contrast to the non-treated control, mice treated with BC-N102 exhibited increase body weight gain, suggesting that in addition to the therapeutic effect on tumor growth, BC-N102 ameliorated the tumor induce loss of body weight and does not mediate obvious adverse effect in the animal.

## Conclusions

Our results demonstrated that BC-N102 suppresses human breast cancer tumorigenesis via time-course and dose-dependent cell cycle arrest at G0/G1 phase, and regulation of cell cycle proteins. In addition, BC-N102 suppresses both ligands mediated (classical) and non-ligand mediated (alternative) pathways of ER, AR, and PR activation while mediating the activation of MR signaling in breast cancer. Our results suggest that the proper application of BC-N102 may be a beneficial chemo-preventive strategy for breast cancer patient.

## Abbreviations

ER: estrogen receptor
PR: progesterone receptor
AR: androgen receptor
PI3K: phosphatidylinositol 3-kinase
p-ERK: phosphorylated extracellular signal-regulated kinase
MAPK: mitogen-activated protein kinase
CDK2: cyclin dependent kinase 2
CDK4: cyclin dependent kinase 4
HER2: human epidermal growth factor receptor 2
mTOR: mammalian target of rapamycin
AKT: protein kinase B
TCGA: The Cancer Genome Atlas
SRB: sulforhodamine B
NSCLC: non-small-cell lung cancer
CNS: central nervous system
TCA: trichloroacetic acid
ChIP: Chromatin immunoprecipitation
MTD: Maximum tolerated dose
PDB: protein data bank
